# Reduced soluble TNF-like weak inducer of apoptosis (sTWEAK) and increased frap in postmenopausal women with metabolic syndrome

**DOI:** 10.1186/1758-5996-7-S1-A144

**Published:** 2015-11-11

**Authors:** Fabio Vasconcellos Comim, Etiane Tatsch, Bruna Hausen, Juliana Wispel, Leo Canterle Dal Osto, Rizzi Adhan de Viera, Paola Roehrs Colpo, Pietra Zorzo, Lucas Venturini Zottele, Karen Koff da Costa, Rafael Noal Moresco, Melissa Orlandin Premaor

**Affiliations:** 1 Universidade Federal de Santa Maria, Santa Maria, Brazil

## Background

Recent evidence in the literature has reported the association between the soluble bioactive form of TNF-like weak inducer of apoptosis (sTWEAK) to insulin resistance, type 2 DM, chronic kidney disease, and atherosclerosis. To date, just a single study has evaluated this novel inflammatory cytokine in Metabolic Syndrome (MetS), showing a reduction in the circulating levels of sTWEAK in elder Caucasian patients (>97% hypertensive) at high cardiovascular risk. Nevertheless, is not known whether individuals from a mixed population at the primary care may exhibit or not similar changes.

## Objectives

The aims of this study were to investigate the association between MetS and biomarkers of chronic inflammation (plasma sTWEAK) and oxidative stress (AOPP, Nox, FRAP) in postmenopausal women recruited at community level.

## Materials and methods

This cross-sectional analysis enrolled 157 women from a previous community-based study of 1057 postmenopausal women covering all basic health units from the municipality of Santa Maria-RS. Participants answered a questionnaire including the clinical history and co-morbidities and were submitted to anthropometric evaluation (height, weight, abdominal circumference), measurement of blood pressure and blood sample collection. Plasma levels of sTWEAK were assessed by ELISA. Biochemical analysis (glucose, LDL, HDL, triglycerides) and oxidative stress markers (AOPP, Nox and FRAP) were determined in serum as previously described. The definition of MetS was based by established criteria of the International Diabetes Federation (IDF). This study was approved by the local Ethics Committee. Assimetric data was logN transformed. The significance level was set at P<0.05.

## Results

No differences in age were observed between postmenopausal women of non-MetS(n=42) and MetS (n=115)groups. The (mean + SD) of age was, respectively, 66.6 + 7 y and 68.6 + 6 y in non-MetS and MetS. The diagnosis of hypertension was present in 17% of non-MetS and 85% of MetS women. Interestingly, FRAP levels were significantly higher in MetS (522.6 + 296 µmol/L) against non-MetS (411.1 + 221 µmol/L)(p=0.02). sTWEAK in plasma was reduced in MetS ( 282.9 + 158 pg/mL) versus non-MetS (338.3 +162 pg/mL) and reach significance after logN transformation (p=0.027).

## Conclusion

Our Results supports for the first time a disruption of (sTWEAK) and oxidative stress marker (FRAP) in postmenopausal women with MetS in a mixed population obtained from a community-based study.

